# Biological roles of filamin a in prostate cancer cells

**DOI:** 10.1590/S1677-5538.IBJU.2018.0535

**Published:** 2019-01-29

**Authors:** Xue-Chao Li, Chuan-Xi Huang, Shi-Kui Wu, Lan Yu, Guang-Jian Zhou, Li-Jun Chen

**Affiliations:** 1 Department of Urology the Fifth Medical Center Chinese PLA General Hospital Beijing China Department of Urology, the Fifth Medical Center, Chinese PLA General Hospital, Beijing, China;; 2 College of Life Science Hebei University Hebei China College of Life Science, Hebei University, Hebei, China;; 3 Laboratory of Medical Molecular Biology Beijing Institute of Biotechnology Beijing China Laboratory of Medical Molecular Biology, Beijing Institute of Biotechnology, Beijing, China

**Keywords:** Prostatic Neoplasms, Filamins, RNAi Therapeutics

## Abstract

**Objective:**

This study aims to investigate the association of filamin A with the function and morphology of prostate cancer (PCa) cells, and explore the role of filamin A in the development of PCa, in order to analyze its significance in the evolvement of PCa.

**Materials and Methods:**

A stably transfected cell line, in which filamin A expression was suppressed by RNA interference, was first established. Then, the effects of the suppression of filamin A gene expression on the biological characteristics of human PCa LNCaP cells were observed through cell morphology, in vitro cell growth curve, soft agar cloning assay, and scratch test.

**Results:**

A cell line model with a low expression of filamin A was successfully constructed on the basis of LNCaP cells. The morphology of cells transfected with plasmid pSilencer-filamin A was the following: Cells were loosely arranged, had less connection with each other, had fewer tentacles, and presented a fibrous look. The growth rate of LNCap cells was faster than cells transfected with plasmid pSilencer-filamin A (P <0.05). The clones of LNCap cells in the soft agar cloning assay was significantly fewer than that of cells stably transfected with plasmid pSilencer-filamin A (P <0.05). Cells stably transfected with plasmid pSilencer-filamin A presented with a stronger healing and migration ability compared to LNCap cells (healing rate was 32.2% and 12.1%, respectively; P <0.05).

**Conclusion:**

The expression of the filamin A gene inhibited the malignant development of LNCap cells. Therefore, the filamin A gene may be a tumor suppressor gene.

## INTRODUCTION

The incidence of prostate cancer (PCa) is increasing globally. In Europe and the United States, PCa has the highest incidence among malignant tumors in men, accounting for 25% of malignant tumors in men; and its mortality rate ranks second in men with malignant tumors (1, 2). Approximately 40.000 American men die from PCa annually (3). Most of the newly diagnosed PCa patients have low-risk or benign tumors (4). However, there are still approximately 20-30% of patients with localized PCa who have high-risk tumors (5).

PCa is caused by the transformation and canceration of prostatic epithelial cells. This is a multi-step, multi-stage process (6, 7). Androgen receptors (ARs) play a key role in the development and growth of the prostate gland (8). It also plays an important role in the growth, survival, apoptosis, metastasis and differentiation of PCa cells (9). Filamin A is a 280kDa cytoskeletal protein, which consists of two large fragments of 170kDa and 110kDa, respectively; and the latter can be divided into two parts: 90kDa and 20kDa (10). The 90kDa part can bind with ARs and affect the chromosomal translocation of the nucleus (11, 12). Savoy et al. confirmed that filamin A could regulate AR Nrdp1 in PCa, and affect the growth and survival of PCa (13). Bismar et al. used 12 molecules screened by proteomics combined with gene chip analysis technology as the combination of candidate molecular markers for PCa progression, which were ordered according to the significance of difference in clinical specimens; and the result revealed that filamin A ranked first (14). The Filamin A gene has also been proven to be associated with PCa metastasis. Sun et al. found that filamin A could inhibit the metastasis and invasion of PCa by regulating the expression of MMP-9 (15). Mooso et al. also verified that the level of filamin A in the nucleus and cytoplasm was correlated to the metastatic ability of PCa (16). Narain et al. considered that Filamin-B instead of filamin A could be used as a biomarker for PCa (17). The degree of damage to PCa is positively correlated with its disease progression. How to effectively delay the conversion of hormone-sensitive PCa to castration-resistant prostate cancer (CRPC) is more important in the overall treatment of PCa (18). The Filamin A gene has been shown to be involved in the development and progression of PCa, and further studies of the biological function of Filamin A may provide a new perspective for the overall treatment of prostate cancer.

In order to reveal the realistic biological function of the filamin A gene, in the present study, a stably transfected cell line, in which the expression of filamin A was suppressed by RNA interference, was first established, and the effect caused by filamin A expression levels on cell characteristics was observed, thereby providing experimental data for further research on the function of the filamin A gene.

### Experimental materials

#### Cell lines and plasmids

Human PCa cell line LNCaP and plasmid pSilencer-filamin A were previously preserved in our Department.

## Major reagents

The RPMI1640 and trypsin were purchased from GIBCO®. The quality fetal bovine serum was purchased from Hyclone® (USA). The calf serum was purchased from Sijiqing Bioengineering Co., Ltd.® (Hangzhou, China). HEPES was purchased from Amersham Life Science®. The cell transfection reagent Lipofectamine 2000 was purchased from Invitrogen® (USA). Dimethyl sulfoxide (DMSO) was purchased from Sigma® (USA). The western blot color kit was purchased from Pierce®. The nitrocellulose membrane was purchased from Bio-Rad®. The filamin A antibody was purchased from Chemicon®. Other reagents were analytical pure products made in China. The eukaryotic transfection reagent Lipofectamine 2000 was purchased from Vigorous Biotechnology®.

## Major instruments

The Trans-Blot SD semi-dry electric transfer apparatus and Steri-cycle carbon dioxide incubator were purchased from Thermo Electron Corporation®. The XDS-1B microscope was purchased from Chongqing Optical Instrument Co. Ltd®. The IX70 fluorescent inverted microscope was purchased from Olympus®. The 3K18 refrigerated centrifuge was purchased from Sigma® (USA). The PCR machine was purchased from Biometra®. TheEL311SX ELISA kit was purchased from BioTek® (US). The clean bench was purchased from Beijing Semiconductor Equipment First Factory®.

## Experimental methods

### Cell cultivation

(1) The preserved cells were taken out from the liquid nitrogen or -80°C refrigerator. The frozen storage tube that contained the cells were rapidly placed in a water bath at 37-42°C, and shaken slightly to promote it to melt.

(2) Cells were gently suspended with RPMI1640 containing 8% fetal bovine serum, and transferred to the centrifuge tube.

(3) Cells were centrifuged at 1.000rpm, and the supernatant was discarded.

(4) Cells were added with 5mL of RPMI1640 containing 8% Hyclone fetal bovine serum, transferred in a cell culture flask, and cultured in an incubator at 37°C with 5% CO2 and saturated humidity.

## Transfection of eukaryotic cells

(1) Cells were first inoculated on six-well plates, and cultured until approximately 75% fusion.

(2) Plasmid DNA was diluted with serum-free RPMI1640 medium, and the Lipofectamine2000 was diluted with the same medium. The above two were mixed and placed at room temperature for 20 minutes.

(3) The cells were transferred to a serum-free medium. After 20 minutes, the mixture was added to the cell medium.

(4) These were converted into normal medium after 4-6 hours.

(5) Cells were cultured in an incubator at 37°C with 5% CO2 and saturated humidity.

(6) Cells were screened by hygromycin until the formation of cell clones could be visually observed. Then, cells were extracted by capillary and subjected to amplification culture.

## Cell morphology

(1) Cells were inoculated in culture dishes at a lower density, and placed in an incubator for culture at 37°C with 5% CO2 and saturated humidity.

(2) The morphological changes in cells were observed, photographed and recorded.

## Western blot

(1) These cells were obtained in the normal state, and the protein was extracted.

(2) After the protein was processed, the absorption value of the optical density was read in the microplate reader at a wavelength of 562nm, and the protein concentration was calculated based on the formula: y=1.1308x+0.0802.

(3) The protein was loaded and processed. Then, coloration, tabletting and development were performed using the chemiluminescence method.

## Tetrazolium salt colorimetry assay

(1) Cells were inoculated in 96-well plates at a density of 2.000 cells/well, and eight duplicated wells were set.

(2) Cells were allowed to stand overnight to adhere to the wall, added with 20μl of tetrazolium salt MTT (5mg/mL dissolved in phosphate buffered solution [PBS]), and cultured in an incubator at 37°C with 5% CO2 and saturated humidity for four hours.

(3) The supernatant was discarded and 150μl of DMSO was added to dissolve it.

(4) The OD490 value was read on the microplate reader at a 490nm wavelength, which was defined at the OD490 value of day one.

(5) Then, the OD490 value was read daily, the ratio of the value of day n to the value of day one was defined at the relative value of day n. Finally, the growth curve was drawn based on the relative value and analyzed.

## Agar colony forming experiment

(1) Preparation of the bottom gel: One volume of 5% agar was mixed with nine volumes of preheated medium, and were poured into six-well plates.

(2) Cells were inoculated on six-well plates according to gradients of 2.000, 4.000 and 6.000 cells/well, and mixed with a certain proportion of agar. Each cell density occupied two wells.

(3) Cells were placed in an incubator and cultured at 37°C with 5% CO2 and saturated humidity for three weeks.

(4) Clones with diameters of >75μM or >50 cells were counted.

## Cell scratch test

(1) Photographing and measurement of the scratch width under a microscope.

(2) Cells were placed in an incubator at 37°C with 5% CO2 and saturated humidity for culture. Then, continuous observation was performed, cells were photographed with a camera, and the width of the scratch was measured after healing.

(3) The scratch repair rate was calculated according to the formula: scratch repair rate= (initial scratch width-scratch width after healing) /initial scratch width ×100%.

## Data analysis

All statistics analysis of the experimental data were processed and analyzed using SAS software. Data obtained from MTT assay and soft agar colony formation experiments were analyzed using one-way analysis of variance. Data obtained from in vitro cell migration experiments were analyzed using the t-test in the SAS software.

### Experimental results

#### The establishment of the PCa cell line where the expression of filamin A was inhibited

**Figure 1:**
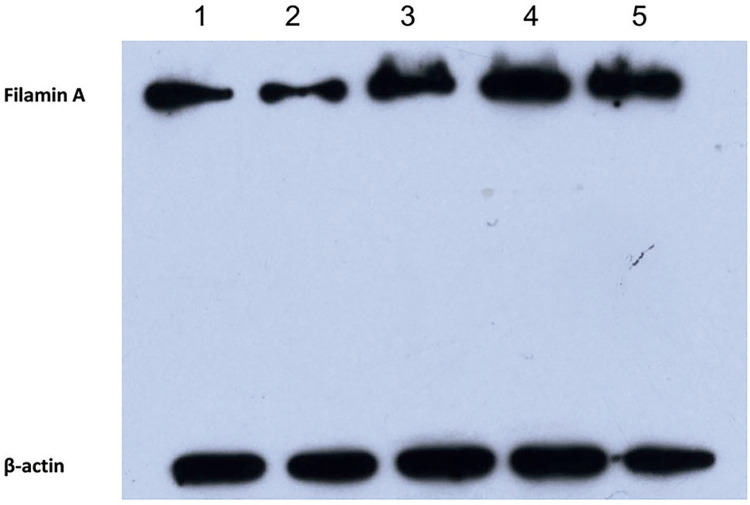
The expression of Filamin A in LNCaP cell line that was transfected with different plasmids by Western-blot. Stably transfected pSilencer-Filamin A recombinant plasmid LNCaP cell line (1, 2). Stably transfected pSilencer-negative recombinant plasmid LNCaP cell line (3, 4). Normal cell line (5). The result of 2 is significant.

The PCa LNCaP cell line was transfected with recombinant plasmids of pSilencer-filamin A and pSilencer-negative, which were screened and identified by hygromycin and Western blot. Then, the PCa cell line, in which the expression of filamin A was effectively inhibited, was obtained (Figure-1).

## Change in cytomorphology

**Figure 2:**
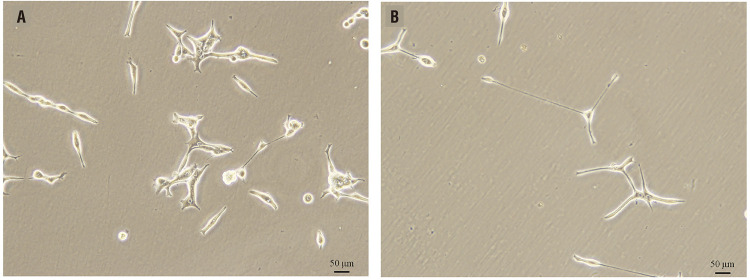
Changes in cell morphology. Morphological characteristics of Normal LNCaP cell line (A). Morphological characteristics of LNCap cell line that transfected pSilencer-Filamin A (B), compared to LNCaP , the cells are loosely arranged, with less contact with each other, fibrous, and with fewer tentacles.

The cells in logarithmic growth phase in each group were observed under light microscopy. These results revealed the following LNCap cell phenotypic characteristics: cells were closely arranged, had rich contact with each other, and were thick and had increased adherent antennae (Figure-2A). The morphology of cells transfected with plasmid pSilencer-filamin A: These cells were loosely arranged with less connection with each other and fewer tentacles, presenting a fibrous look (Figure-2B).

## In vitro growth curve of cells

**Figure 3:**
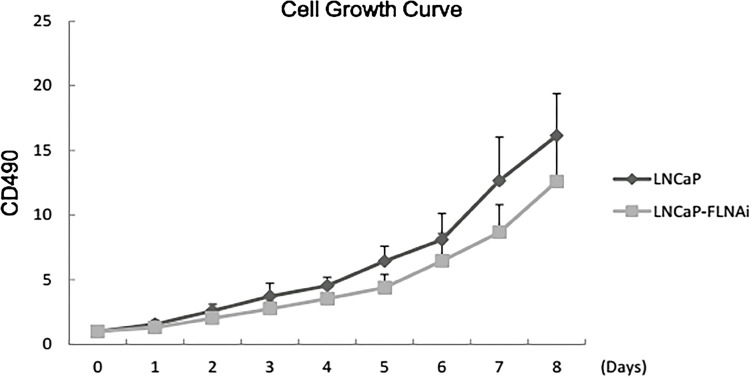
Observe the growth status in vitro by MTT assay. The experimental results show that the growth of LNCaP cells (Blue) is faster than cells transfected with pSilencer-Filamin (Pink, p <0.05), fifth and seventh days, the growth rate of LNCaP cells is 1.5 times of cells transfected with plasmid pSilencer-Filamin A. Filamin A gene expression inhibits cell division and growth process.

In order to investigate the effect of the expression of filamin A gene on the proliferation of LNCap cells, the in vitro growth of LNCap cells and cells transfected with plasmid pSilencer-filamin A were observed by tetrazolium salt colorimetry assay. In order to eliminate the error caused by the cell count, the count of cells on day zero was set as 100%, and the relative number of cells in each group on each day (%) was calculated respectively. That is, the relative cell number (%) = the OD value on day n/ the OD value on day 0×100 (n=1, 2, 3). With time as the abscissa and the relative cell number (%) as the ordinate, the cell growth curve was drawn (Figure-3). The results of this test revealed that the growth rate of LNCap cells was significantly faster than of cells transfected with plasmid pSilencer-filamin A, and difference was statistically significant (P <0.05). The growth rate of LNCap cells on day five and seven was 1.5 times faster than that of cells transfected with plasmid pSilencer-filamin A. Under the same culture condition, the number of proliferated cells transfected with plasmid pSilencer-filamin A was less than that of LNCap cells. This appears to be in contradiction with the phenomenon in our clinical practice, in which the higher the malignant degree is, the larger the tumor tissue in the PCa patient becomes.

## Soft agar cloning assay

The anchorage-independent growth ability of cells on soft agar is an important indicator to measure the malignant degree of cells under the condition of in vitro culture. Therefore, the ability of these two groups of cells to grow on soft agar was measured. Under a low magnification microscope, the number of clones with more than 50 cells was counted, and the effect of different inoculation densities on cloning efficiency was compared. These results reveal that no matter how many cells were inoculated, the clones of LNCap cells in the soft agar cloning assay was significantly fewer than that of cells stably transfected with plasmid pSilencer-filamin A, and the difference was statistically significant (P <0.05). When these two groups of cells were inoculated at densities of 2.000, 4.000 and 6.000 cells/well, the clones of the cells stably transfected with plasmid pSilencer-filamin A in the soft agar cloning assay increased by 3.1, 2.7 and 3.8 times, compared with LNCap cells, respectively. This suggests that the inhibition of the filamin A gene can significantly promote the cloning of LNCap cells in vitro.

## Scratch test

**Figure 4:**
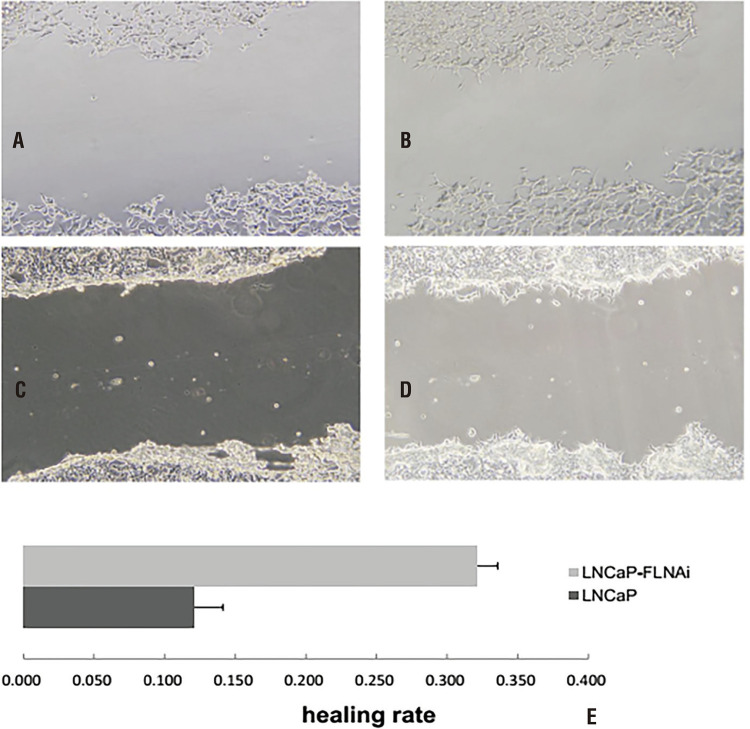
Wound Healing of Cells transfected plasmid pSilencer-Filamin A. LNCaP cell lines transfected plasmid pSilencer-Filamin A with 0 hours (A). LNCaP cell lines transfected plasmid pSilencer-Filamin A with 48 hours (B). Wound Healing of LNCaP cell lines with 0 hours(C). Wound Healing of LNCaP cell lines with 48 hours (D). Stable transfected plasmid pSilencer-Filamin A cells had higher migration ability than LNCap cells (E, healing rates were 32.2% and 12.1%; p <0.05)

The scratch test is a common method for measuring cell migration ability. The scratch healing ability of cells in these two groups under the same conditions was determined by observation, photography and measurement using a fluorescence inverted microscope. These results revealed that cells stably transfected with plasmid pSilencer-filamin A presented with stronger healing and migration ability than LNCap cells (healing rates were 32.2% and 12.1%, respectively; Figure-4), and the difference was statistically significant (P <0.05). This suggests that the inhibition of the filamin A gene can significantly strengthen the migration ability of LNCap cells in vitro. This indicates that the malignant degree of LNCap cells transfected with plasmid pSilencer-filamin A was elevated.

## DISCUSSION

In the present study, the PCa LNCaP cell was chosen as the study subject. The LNCaP cell is an androgen-dependent (AD) PCa cell, and is equivalent to the early stage of clinical PCa. A study reported that on the basis of LNCaP cells, a sub cell line, androgen-independent (AI) PCa cell line C4-2, has been developed. On this basis, the occurrence and development of clinical PCa were constructed by simulation: ADPCa→AIPCa (19, 20). At present, the LNCaP cell line has become an effective model for studying PCa, the process of PCa progressing into AI, and the occurrence of metastasis (3).

After the LNCap cell line was transfected with plasmid pSilencer-filamin A, the expression of the filamin A gene was inhibited, which is consistent with previous literature (21-23). Compared to LNCaP cells, the morphology of these was characterized as follows: cells were loosely arranged, had less connection with each other, had fewer tentacles, and presented a fibrous look. These coincide with the basic characteristics of cancer cells, that is, the adhesion of cancer cells decreases, and cancer cells lose contact inhibition on growth. This trend increases with the increase in the malignant degree of cancer cells. In these experimental results, it was manifested as that with the increase in the malignant degree of PCa cells, the degree that cancer cells lose contact inhibition on growth increased.

Cell proliferation ability is an important index to determine cell viability. In the present study, the in vitro growth curves of LNCap cells and cells transfected with plasmid pSilencer-filamin A were determined by MTT colorimetry assay. The results revealed that under the same culture condition, the number of proliferated cells transfected with plasmid pSilencer-filamin A was less than that of LNCap cells. This seems to be inconsistent with the perception that the degree of malignancy of the tumor is positively correlated with the growth rate of the tumor cells and the ability to spread. In fact, the reason for the slower growth rate of the plasmid pSilencer-filamin A transfected LNCap cells was significantly associated with decreased expression of filamin A. Studies have shown that filamin A is one of the substrates for CDK1 binding (24). CDK1 regulates the cell cycle and determines whether the cell cycle enters the cell division phase from the intercellular phase (25, 26). A significant decrease in filamin A expression affects the cell cycle, resulting in pSilencer-filamin A transfected LNCap cells. At the same time, filamin A is an important actin cross-linking protein that is involved in actin rearrangement. Before cell division, the morphology of the cells is deformed, the cytoskeleton rearranges, and the microfilaments composed of actin are involved. Filamin A may affect the cell cycle by affecting the rearrangement of the cytoskeleton (27, 28). It is well-known that some limitations exist in the simulation of in vivo growth of tumor cells in cell experiments in vitro, since the growth environment of tumor cells in vivo is very different from the experimental environment of in vitro cultivation. The soft agar cloning assay can reflect the population dependence and proliferation ability of cells, and is more similar to the internal environment, compared with cells simply cultured in the culture dish with a culture medium. The cloning efficiency of cells was positively correlated with the malignant degree of cells, which is a common basis for detecting tumor cells. These results reveal that no matter how many cells were inoculated, the clones of cells stably transfected with plasmid pSilencer-filamin A in the soft agar cloning assay were significantly more than that of LNCap cells, and the anchorage-independent growth ability was enhanced; that is, the malignant degree increased.

When tumor cells separate from the mother tumor cells, cross the vessel wall and invade surrounding normal tissues, it requires cells to have certain movement ability (29, 30). Highly metastatic tumor cells usually have strong motility. In the present study, the scratch test was used to compare the movement ability of LNCap cells transfected with pSilencer-filamin A and untransfected LNCap cells. The scratch healing ability of cells in these two groups under the same condition was determined. These results reveal that cells stably transfected with plasmid pSilencer-filamin A presented with stronger healing and migration abilities than LNCap cells. This suggests that the malignant degree of LNCap cells transfected with plasmid pSilencer-filaminA increased.

The degree of harm of PCa is positively correlated with the progression of the disease. Once entering the CRPC stage, the condition of patients often deteriorates rapidly within a short time. Finding effective therapeutic targets before the emergence of CRPC is bound to play a key role in preventing the progression of disease. The inhibition of the expression of the filamin A gene increases the malignant degree of LNCap cells. From this, we can speculate that the filamin A gene may be a tumor suppressor gene, and play an important role in the occurrence and development of tumors, especially in PCa. Furthermore, it may become a new molecular marker for the occurrence and development of PCa. In hormone sensitive stage PCa, if adding filamin A gene expression, or reduce inhibition of filamin A gene expression will reduce hormone sensitivity prostate proliferation, transfer ability, and slow down the transformation process of PCa from hormone sensitive phase to CRPC phase, will bring benefit to the overall treatment in patients with PCa. In summary, this study provides an experimental and theoretical basis for pre-clinical studies on gene diagnosis and gene therapy of PCa. These are the primary explorations on the function of the filamin A gene in PCa cells. In future studies, we will further investigate the expression of the filamin A gene at the PCa tissue level.
